# Mitochondria Retrograde Signaling and the UPR^mt^: Where Are We in Mammals?

**DOI:** 10.3390/ijms160818224

**Published:** 2015-08-06

**Authors:** Thierry Arnould, Sébastien Michel, Patricia Renard

**Affiliations:** 1 Laboratory of Biochemistry and Cell Biology (URBC), Namur Research Institute for Life Sciences (NARILIS), University of Namur, Rue de Bruxelles 61, 5000 Namur, Belgium; E-Mails: sebastien.michel@unamur.be (S.M.); patsy.renard@unamur.be (P.R.); 2 Department of Physiology, University of Lausanne, Rue du Bugnon 7, CH-1005 Lausanne, Switzerland

**Keywords:** mitochondria, unfolded protein response, cell signaling, gene expression

## Abstract

Mitochondrial unfolded protein response is a form of retrograde signaling that contributes to ensuring the maintenance of quality control of mitochondria, allowing functional integrity of the mitochondrial proteome. When misfolded proteins or unassembled complexes accumulate beyond the folding capacity, it leads to alteration of proteostasis, damages, and organelle/cell dysfunction. Extensively studied for the ER, it was recently reported that this kind of signaling for mitochondrion would also be able to communicate with the nucleus in response to impaired proteostasis. The mitochondrial unfolded protein response (UPR^mt^) is activated in response to different types and levels of stress, especially in conditions where unfolded or misfolded mitochondrial proteins accumulate and aggregate. A specific UPR^mt^ could thus be initiated to boost folding and degradation capacity in response to unfolded and aggregated protein accumulation. Although first described in mammals, the UPR^mt^ was mainly studied in *Caenorhabditis elegans*, and accumulating evidence suggests that mechanisms triggered in response to a UPR^mt^ might be different in *C. elegans* and mammals. In this review, we discuss and integrate recent data from the literature to address whether the UPR^mt^ is relevant to mitochondrial homeostasis in mammals and to analyze the putative role of integrated stress response (ISR) activation in response to the inhibition of mtDNA expression and/or accumulation of mitochondrial mis/unfolded proteins.

## 1. Introduction

Mitochondrial, multifunctional, and dynamic organelles (resulting from fusion and fission events of mitochondria fragments, according to cell type and conditions) are linked to pathologies far beyond the *sensu stricto* “mitochondrial diseases” because they regulate metabolism via the Krebs cycle and oxidative phosphorylation (OXPHOS). Linked to their bioenergetics, mitochondria that contain more than 1000 different proteins/peptides, synthesized from both mitochondrial and nuclear genomes in a coordinated manner [1,2] also participate in synthesis (steroids, amino acids, nucleotides), the biogenesis of iron-sulfur centers, calcium homeostasis and redox status [3], control epigenetics marks in the nuclear genome, and constitute an integration platform for signaling and cell death/survival signals, connecting the organelle to apoptosis and autophagy [4,5,6,7]. The continued organelle maintenance can be seen as a balance between the biogenesis of the organelle and the mechanisms that provide quality control (involved in the remodeling and mitophagy) that guarantees cell homeostasis and function [8,9]. This function is maintained by the participation of several chaperones, antioxidant enzymes (SOD2, PRDX3, PRDX5, GPX1: human nomenclature according to Uniprot), and quality control proteases that promote protein folding and stability on the mitochondria while performing the degradation of un- or mis-folded proteins that accumulate [10]. Essential for this control is the molecular communication between the mitochondria and nucleus, in which ATP, calcium, and reactive oxygen species have been described to play major roles [1,11,12,13,14]. Indeed, upon organelle dysfunction, which can be caused by many events, such as mtDNA depletion, mutations, deletions, oxidative stress, aggregation of misfolded proteins, or dramatic changes in morphology and dynamics, a mitochondrial-to-nucleus communication, known as “retrograde signaling,” triggers an orchestrated expression of nuclear genes in an attempt to relieve/resolve the stress and/or to compensate the defect [15]. The importance of these stress response pathways can be highlighted by the various pathophysiological developments associated with their impairment [15]. The aim of the current review is to highlight recent developments in the role of mitochondrial unfolded protein response (UPR^mt^) in the field of mitochondrial dysfunction and putative connection with other retrograde signaling pathways, with a particular focus on differences between mechanisms retrieved in mammals and other organisms.

### 1.1. Mitochondrial Retrograde Responses

Several signaling pathways have been described in the mitochondria-to-nucleus communication observed in response to organelle stress and dysfunction. This mitochondrial stress response can be perceived as an attempt to compensate for the metabolic defect by stimulating several biological processes including mitochondria biogenesis. The transcriptional regulation of mitochondrial biogenesis is mediated by a set of transcription factors such as NRF1 and NRF2/GA-binding protein subunit β-1 (nuclear respiratory factors 1 and 2), cyclicAMP-responsive element binding protein1 (CREB1), steroid hormone receptor ERR1 (ERRα), and PPARG (PPARγ). The activity of these transcription factors is coordinated by members of the PGC-1 co-activator family [16]. The retrograde communication often relies on some of these transcriptional regulators to modulate gene expression to adapt mitochondria function in response to the original cue and promote recovery and function to resolve the stress. We now discuss the signaling pathways that regulate the expression, localization, and activity of these transcription factors. Notably, if the following pathways are usually associated with the regulation of mitochondrial protein abundance, lipid content might also be regulated and controlled by retrograde signaling. As with the absence of mtDNA in HeLa cells, mitochondrial DNA absence sensitive (MIDAS) factor increases mitochondrial mass by regulating the expression of enzymes involved in cardiolipin synthesis [17].

Retrograde communication in response to mitochondrial stress comes from seminal studies from Butow’s group in the budding yeast *Saccharomyces cerevisiae* depleted of mtDNA [18,19], which showed that electron transport chain (ETC) deficiency leads to the transcription of genes associated with glutamate metabolism (reviewed in [20]). In yeast, there are three retrograde response genes (RTG): Rtg1p and Rtg3p are transcription factors, forming a dimer that translocates from the cytosol to the nucleus to regulate gene expression, while Rtg2p would act/behave as a sensor of mitochondrial stress [21]. Although mammalian orthologs of these proteins have not been found, similar signaling pathways do exist in mammals, as detailed in the next section.

#### 1.1.1. Retrograde Signaling Involving Increased Cytosolic Calcium Concentration

Pioneering works on retrograde signaling in mammalian cells have been described in mtDNA-depleted cells through the pharmacological inhibition of ETC complexes or disruption of the mitochondrial membrane potential and focused on calcium in mouse C2C12 myocytes and human pulmonary carcinoma A549 cells. Avadhani’s group showed that mitochondrial dysfunction associated with membrane depolarization leads to increased cytosolic calcium concentration and triggers nuclear factor of activated T-cells (NF-AT) and activating transcription factor 2 (ATF2) translocation to the nucleus in a calcineurin (Cn)- and Mitogen-activated protein kinase 8 (MAPK8)/JNK1-dependent manner, respectively [11,22]. The list of transcriptional regulators activated by calcium, either by a phosphorylation in a JNK1- or calcium/calmodulin-dependent protein kinase type IV (CaMKIV)-dependent manner, or by a dephosphorylation mediated by calcineurin in response to mitochondrial dysfunction has now expanded and includes the transcription factors CREB1, nuclear factor of κ light polypeptide gene enhancer in B-cells 1 (NF-κB), cellular tumor antigen p53 (p53), myocyte-specific enhancer factor 2A (MEF-2), and co-activators peroxisome proliferator-activated receptor γ coactivator 1-α (PGC-1α) and heterogenous nuclear ribonucleoprotein A2 (hnRNPA2) [13,22,23,24,25]. Altogether, data about calcium-dependent mitochondrial retrograde response represent a good example of functional compensation after organelle stress. Overexpressed nuclear genes include proteins associated with calcium homeostasis, such as calsequestrin and calreticulin, OXPHOS subunits for stabilizing energy production, and chloride intracellular channel protein 4 (Clic 4), which contribute to mitochondrial membrane potential [26,27,28].

#### 1.1.2. Retrograde Response Associated with ROS Production and Signaling

Reactive oxygen species (ROS) are by-products of ETC complexes, and while they represent important physiological second messengers, their excessive production can lead to protein, lipid, and DNA damages and, ultimately, cell death. When antioxidant defenses are overwhelmed, a transcriptional program regulated by the NRF2/GA-binding protein subunit β-1 is initiated [29]. Indeed, excessive production of ROS triggers the stabilization and translocation of the factor to the nucleus, where the transcription of target genes containing an antioxidant-response element (ARE) in their promoter is increased, including NRF1, which regulates mitochondrial gene expression [29,30]. It has also been suggested that NRF1 might be directly regulated by redox signaling in phosphatidylinositol 3-kinase (PI3K) and RAC-α serine/threonine-protein kinase/Akt-dependent manners to regulate mtDNA transcription and replication by increasing mitochondrial transcription factor A (TFAM) protein expression [31]. PGC-1α is also activated by AMP-activated protein kinase (AMPK) in response to mitochondrial ROS to promote organelle biogenesis [32]. As for calcium, ROS have been the focus of numerous studies on retrograde communication, and additional transcription factors, such as NF-κB, p53, and AP-1, are activated by oxidants [33,34,35]. Surprisingly, while several studies have demonstrated enhanced biogenesis in response to mitochondrial stress [33,36,37,38], recent biological analysis of retrograde signaling by Chae and collaborators demonstrated the role of ROS in the down-regulation of OXPHOS enzymes [39]. These authors reported that, among the 72 transcription factors, for which activity was modified in response to mtDNA mutation, the abundance of RXRα Retinoic acid receptor RXR α) decreased after ROS-dependent JNK1 activation. As a direct consequence, the interaction between RXRα and PGC-1α was reduced, which attenuated the expression of gene encoding of OXPHOS enzymes as well as mitochondrial ribosomal proteins—two phenomena that further exacerbate mitochondrial dysfunction [39].

#### 1.1.3. Energy Deprivation and Retrograde Response

The most obvious retrograde signaling might be the stress response associated with energy deprivation. AMPK is a sentinel activated in response to an increase in the AMP/ATP ratio, which triggers allosteric activation and the serine/threonine-protein kinase STK11 (LKB1)-dependent Thr172 phosphorylation of AMPK. Once activated, AMPK increases ATP production by inducing mitochondrial biogenesis, thus stimulating fatty acid β-oxidation and glycolysis, and shuts down energy consuming processes such as cell growth and lipid and protein synthesis [40,41,42,43]. AMPK regulates PGC-1α activity and abundance as well as its binding activity to transcription factors, such as NRF1, at the promoter of nuclear gene encoding mitochondrial proteins [36,42]. Moreover, AMPK is also well-known for regulating the serine/threonine-protein kinase mammalian target of rapamycin (mTOR) through control of tuberous sclerosis 2 (Tuberin) activity [44]. In mammals, two complexes have been described: mTORC1 and mTORC2. While mTORC1 regulates processes, such as transcription, translation, autophagy, and metabolism, mTORC2 is mainly associated with cell survival, proliferation, and metabolism. The activities of mTOR complexes are negatively regulated by Hamartin/Tuberin (tuberous sclerosis 1/2) via phosphorylation. In response to mitochondrial stress and energy deprivation, AMPK decreases mTOR activity and downstream processes, such as protein synthesis, by a mechanism involving either direct phosphorylation of the raptor or the activating phosphorylation of Tuberin [41,43,44].

Finally, the NAD^+^/NADH ratio and acetyl-coenzyme A (acetyl-CoA) are important mitochondrial coenzymes and metabolites, respectively, which are associated with retrograde response [45]. However, the reduction of NAD^+^ into NADH during the Krebs cycle and its oxidation by the NADH dehydrogenase complex represent the basis of mitochondrial respiration. Because NAD^+^ is also a substrate used by enzymes, such as poly[ADP-ribose] polymerase-1 (PARP-1) and Sirtuins, the NAD^+^/NADH ratio, which not only results from the redox status of the molecule, but also a balance between its synthesis and its degradation, might modulate gene expression [46]. Conversely, acetyl-CoA, resulting from pyruvate metabolism and fatty acid β-oxidation, is a substrate for lysine acetylation by acetyltransferases that controls the acetylation status of the transcription factors of the forkhead box protein O family members [47], histone acetylation, chromatin remodeling, and thus gene expression [48,49]. Yet, even if both NAD^+^/NADH and acetyl-CoA are associated with the activity of post-translational modifying enzymes, and thus regulation of signaling pathways or gene expression, their direct involvement in retrograde signaling has not been extensively characterized to date.

#### 1.1.4. Beyond Regulation by Transcription Factors

Beyond this level of retrograde communication, which regulates nuclear gene expression by the activation of transcription factors, it has been shown that the mtDNA copy number can also lead to epigenetic modifications. It is known that ATP, acetyl-CoA concentration, NAD^+^/NADH ratio, and SAM (s-adenosylmethionine) are cofactors and metabolites whose concentrations can be modulated by mitochondria. Several of them, such as acetyl-CoA and SAM, are important acetyl- or methyl-group donors for DNA, RNA, and protein modifications that could also control gene expression and contribute to adaptation to mitochondria dysfunction [50]. These molecules can thus be seen as messengers for retrograde signaling responses [50].

Indeed, it has been demonstrated that mitochondrial depletion leads to increased promoter methylation of nuclear genes, which can be reverted, at least partly, by mtDNA repletion [7]. In addition, in conditions of mitochondrial stress, mitochondria-to-nucleus signaling is also know to confer a long-term adaptive response to the cell, which can protect it from future insults related or not to the initial stress. This concept, called mitochondrial hormesis or mitohormesis, provides a long-term increase in cytoprotective function after mild and transient mitochondrial stress such as ROS production. Mitohormesis has been particularly highlighted in the context of increased lifespan in various organisms such as yeast, worms, flies, and mice [51]. Moreover, it has been suggested that mitochondrial stress can generate a non-cell-autonomous response, meaning the activation of a stress response in a cell/tissue that is not affected by the initial triggered stress event. For instance, in response to OXPHOS deficiency, muscle fibers secrete the cytokine fibroblast growth factor 21 (FGF21) in the bloodstream, which results in chronic lipid recruitment and mobilization from adipose tissue and triggers ketogenesis in the liver [52,53]. It seems that skeletal muscle increases FGF21 expression in mitochondrial disorders to compensate for metabolic insufficiency by activating the mTOR Transcriptional repressor protein YY1-PGC-1α pathway [54]. Durieux and co-workers have described another example in *C. elegans*, in which stressed mitochondria, via electron chain manipulations in key tissues, release soluble signals (the so-called mitokines), which trigger expression of cytoprotective genes in distal tissues and have a beneficial effect on longevity of the worm [55]. Mitokines have also been unveiled in the context of a recent retrograde response, which we discuss in the next sections of this review: the mitochondrial unfolded protein response (UPR^mt^). Following the example of the endoplasmic reticulum unfolded protein response (UPR^er^), when damaged and/or unfolded proteins accumulate within mitochondria, this stress response coordinates expression of chaperones and proteases in a positive feedback loop and results in a first attempt to resolve the stress and bring cytoprotection [56].

### 1.2. The Mitochondrial Unfolded Protein Response (UPR^mt^)

Protein homeostasis relies on the equilibrium between the number of unfolded proteins and the folding capacity of a compartment. The mitochondria possess their own arsenal of chaperones and proteases. When unfolded, misfolded, or unassembled proteins accumulate beyond folding capacity, this leads to damage and organelle/cell dysfunction. As seen previously, mitochondrion is able to communicate with the nucleus in response to organelle dysfunction and bioenergetic impairment. As with the endoplasmic reticulum, for which the different signaling pathways and branches of the unfolded protein response are well described [57], and affecting the regulation of autophagy and the biology of mitochondria [58,59], a specific UPR^mt^ could also be initiated to boost folding and degradation capacity in response to unfolded and aggregated protein accumulations in mitochondria. Thus, mitochondrial protein homeostasis—or proteostasis—is preserved by retrograde communication to coordinate transcriptional activation of nuclear-encoded mitochondrial chaperones and proteases. Although its existence was initially reported in mammals almost 20 years ago [60], later studies related to the UPR^mt^ mainly focused in *Caenorhabditis elegans*, with mechanisms mainly revealed in the model organism and linked to aging, healthy lifespan, and longevity [61,62,63]. The UPR^mt^ is now considered as a mechanism that allows synchronization of nuclear and mitochondrial genomes [64].

#### 1.2.1. The UPR^mt^ in *C. elegans*

##### Stress Models for the Study of UPR^mt^

The worm *C. elegans* has been a useful model to identify stresses that activate a UPR^mt^ and to decipher signaling from proteotoxic stress to expression of stress-responsive genes. Most of the time, the induction of the UPR^mt^ can be easily assayed in worms that express green fluorescent protein (GFP) under the control of the promoter that drives the expression of gene encoding chaperones and orthologues of the mammalian mtHSP70 and heat shock 60 kDa protein 1 (HSPD1), respectively [61,62,63]. Ethidium bromide, an inhibitor of the mitochondrial DNA polymerase γ (POLG) was the first UPR^mt^ stressor described in *C. elegans* [65]. Because mitochondrial ETC complexes rely on a precise stoichiometry between nuclear- and mtDNA-encoded proteins, it is reasonable to think that the inhibition of mitochondrial transcription and replication by ethidium bromide, as transcriptions in mitochondria, clearly depend on mtDNA replication [66], which will lead to a protein imbalance and an increase in unassembled components. Similar mechanisms were later used to induce a UPR^mt^ with other molecules. On the one hand, decreasing mtDNA-encoded proteins through interference with mitochondrial translation, either with inhibitors, such as doxycycline, or with siRNA, allowing the silencing of mitoribosomal proteins, triggers a UPR^mt^ [46]. On the other hand, the stimulation of organelle biogenesis, by NAD^+^ supplementation or mTOR inhibition by rapamycin, has similar effects [67]. Additional stresses, which induce a UPR^mt^, following impaired assembly of mitochondrial complexes, defective protein folding, or processing, have also been described in a genome-wide RNAi screen [65].

##### Signaling Pathway of the UPR^mt^

Ten years of research using the UPR^mt^ reporter in worms and large-scale experiments with siRNA were needed to shed light on a currently well-accepted model of retrograde signaling that followed mitochondrial proteotoxic stress in *C. elegans*. Briefly, accumulating unfolded proteins within mitochondria are degraded by the ATP-dependent Clp protease proteolytic subunit (ClpP) into small peptides, which are actively exported across the inner mitochondrial membrane (IMM) by HAF-1, a matrix peptide exporter belonging to the ATP-binding cassette (ABC) transporters [68,69]. Passive diffusion then brings peptides through the outer mitochondrial membrane (OMM) to the cytosol that triggers nuclear translocation of activating transcription factor associated with stress-1 (ATFS-1), a transcription factor that orchestrates expression of mitochondrial chaperones and proteases as well as other genes associated with ROS detoxification, mitochondrial protein import, and glycolysis [70]. Experiments in which the expression of ClpP and HAF-1 was silenced confirmed that both proteins were essential for the nuclear translocation of ATFS-1 and the activation of a UPR^mt^ [68,69]. The mechanism that controls the cellular localization of ATFS-1 was recently discovered [70]. In addition to its nuclear localization sequence (NLS), the transcription factor also contains a mitochondrial targeting sequence (MTS). Under basal conditions, ATFS-1 is constitutively imported in mitochondria and degraded by the Lon protease [71]. However, upon mitochondrial proteotoxic stress conditions, mitochondrial import efficiency decreases, and a fraction of ATFS-1 proteins accumulates in the cytosol and translocates to the nucleus. Very recently, Haynes and colleagues showed that the ATFS-1 transcription factor not only induces chaperones, OXPHOS assembly factor, and glycolysis genes, but also directly regulates OXPHOS gene promoters by limiting the accumulation of OXPHOS transcripts. Altogether, ATFS-1 coordinates the abundance of transcripts involved in OXPHOS expression and assembly factors to the protein-folding capacity of mitochondria [72]. The importance of the efficiency of the import machinery of mitochondrial proteins in the re-localization of ATFS-1 is supported by the fact that the disruption of mitochondrial import in response to the silencing of the mitochondrial import inner membrane translocase subunit Tim23 is sufficient to trigger ATFS-1 accumulation in the nucleus. It can also activate the GFP reporter construct driven by the promoter of HSPD1 encoding HSP60 [70], a mitochondrial matrix chaperonin crucial for the folding and assembly of newly imported mitochondrial proteins [73].

Interestingly, Rainbolt and colleagues obtained similar results and showed an increase in the expression of HSP60-GFP in response to the silencing of Tim17, a response that can be prevented in ATFS-1 but not HAF-1 mutant worms [74]. In addition, these authors noted that silencing of either Tim23 or Tim17 conferred increased resistance to paraquat-induced oxidative stress, although the protection was rather modest in mammalian cells. However, increased resistance to oxidative stress was not disrupted in the ATFS-1 mutant worm, thereby questioning the active participation of the UPR^mt^ in this protection. Finally, while the reduced protein import was able to induce stress-responsive genes independently of mitochondrial matrix stress (HAF-1 mutant), in the context of a UPR^mt^, both the reduced mitochondrial protein import and the ATFS-1 nuclear translocation relied on HAF-1 and peptides efflux; however, the precise mechanism and tight regulation are still unclear [70].

In addition to ATFS-1, in *C. elegans*, the transcription factors DVE-1 and ubiquitin-like protein 5 (UBL-5) appear to be involved in the transcriptional regulation of the UPR^mt^. During the onset of mitochondrial stress, both proteins redistribute into the nucleus and form a complex that binds the promoter of gene encoding chaperones such as mtHSP70 and HSPD1. As for ATFS-1, ClpP is also required for DVE-1 nuclear translocation, but HAF-1 would be dispensable [68]. While these proteins might help in chromatin remodeling to facilitate ATFS-1 access to the promoters of the specific target genes of the UPR^mt^, future research on the importance of the contribution of DVE-1/UBL-5 complex to the UPR^mt^ is still needed. In addition, it is also very likely that the activation of a UPR^mt^ could be accompanied by signals that trigger and connect the activation of other cell signaling pathways.

##### Crosstalk between the UPR^mt^ and Other Stress Pathways in *C. elegans*

The UPR^mt^ relies on more than the over-expression of chaperones and proteases to maintain mitochondria proteostasis; several genes that are directly or indirectly controlled by ATFS-1 have been identified by comparing the relative abundance of transcripts between wild type and ATFS-1 mutant worms raised either under basal or UPR^mt^-inducing conditions. In these conditions, among 685 genes differentially expressed in response to the UPR^mt^, only 391 genes (encoding important components of ROS detoxification and antioxidant enzymes, glycolytic enzymes, and proteins of the mitochondrial protein import machinery) seemed to be dependent on ATFS-1, suggesting the activation of other pathways [70].

Other studies described the UPR^mt^ as interconnected cell signaling with other stress-activated pathways such as the integrative stress response (ISR) [75] and the antioxidant response [67]. Baker and co-workers demonstrated that, during mitochondrial stress, eIF2α is phosphorylated by GCN2 in a ROS-dependent manner, attenuating the protein synthesis within the cytosol. This is similar to what happens in the well-characterized UPR^er^ [75]. These two pathways are complementary and maintain the activity of mitochondria. These authors show that, in a worm genetic model of UPR^mt^, RNAi-mediated silencing of eukaryotic translation initiation factor 2-α kinase 4/GCN2-like protein (GCN2) is associated with the accumulation of carbonylated proteins and decreased oxygen consumption, despite enhanced activity of the mtHSP70-GFP reporter plasmid. Conversely, upon mitochondrial stress, the inhibition of the UPR^mt^ increases the load on the ISR, as demonstrated by an increased eIF2α phosphorylation. In addition, concomitant alterations of both pathways result in growth defects, even in the absence of any stress [75].

In addition, boosting the NAD^+^ level not only activates Sirtuins and, especially, mitochondrial Sirtuin 3 [76], but also induces mitonuclear protein imbalances and activates UPR^mt^ [67]. Importantly, these authors revealed that, in mice and *C. elegans*, in time-course experiments, treatments with nicotinamide riboside (NR), a NAD^+^ precursor led to a protective response in a two-step process. First, during the early phase response (one day of treatment), the UPR^mt^ was activated, with the associated increase in ClpP and HSP6 expression. Then, after three days of treatment with NAD^+^ booster/NR, with the UPR^mt^ markers still overexpressed, an antioxidant response was also triggered, as demonstrated by enhanced activation of the promoter of the gene encoding SOD3 and nuclear localization of the transcription factor daf-16 (FOXO3A orthologue in mammals). Interestingly, it appears that both pathways might be interconnected; the induction of Extracellular superoxide dismutase [Cu/Zn] (SOD3) was dependent on UBL-5, a transcriptional regulator activated during the UPR^mt^ [67].

#### 1.2.2. The UPR^mt^ Models in Mammals

**Figure 1:**
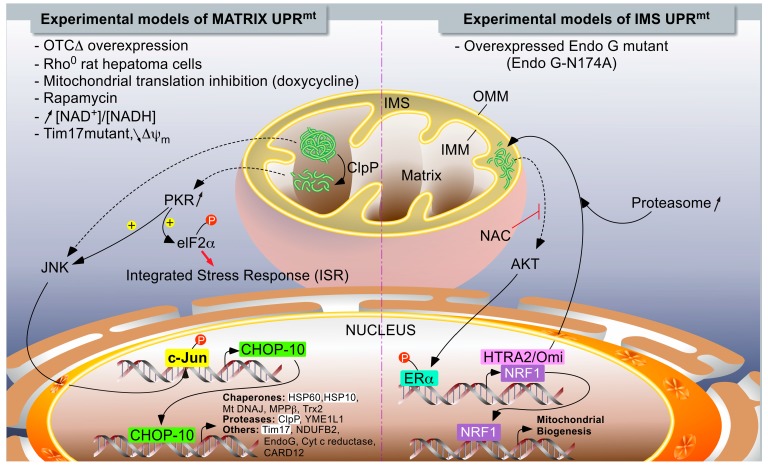
The UPR^mt^ models in mammalians. Two major independent models of UPR^mt^ have been described. When unfolded proteins accumulate in the mitochondrial matrix, they are first cleaved by ClpP proteases. Peptides exit mitochondria by unknown mechanisms and trigger a signaling pathway, leading to the activation of c-Jun N-terminal kinase (JNK) and PKR, which phosphorylate as c-Jun (part of AP-1 transcription factor) and eIF2α, respectively. The phosphorylation of eIF2α can also be mediated by GCN2 in response to mitochondrial translation inhibition (not illustrated), and it turns on the integrated stress response (see Figure 2). PKR could also activate JNK. The activation of c-Jun triggers CHOP-10 expression. In turn, the transcription factor regulates the expression of stress-resolving genes such as mitochondrial proteases and chaperones (left side of the chart). When unfolded proteins accumulate in the inter-membrane space, the UPR^mt^ is mediated by the activation of ERα, triggered by an AKT-dependent phosphorylation. The activation of AKT would then be mediated by oxidative stress, as inhibited by *N*-acetylcysteine (NAC). Mitochondrial stress resolves by an increase in the expression of HTRA2/Omi protease and enhanced proteasome activity, while the biogenesis of mitochondria is enhanced and under the control of NRF1, a target gene of ERα. The highlighted acronyms represent genes for which an endogenous differential expression (RT-qPCR or Western blot analysis) in response to UPR^mt^ has been experimentally demonstrated to be dependent on CHOP-10; this is in addition to analysis using reporter constructs. The non-highlighted genes refer to genes controlled by CHOP-10 in response to a UPR^mt^, but only demonstrated using reporter constructs.

In mammals, two different models of UPR^mt^ have been described (Figure 1). While Hoogenraad’s group described a DDIT3 (DNA damage-inducible transcript 3 protein)/CHOP-10-dependent UPR^mt^ induced by the overexpression of a truncated form of a mitochondrial matrix enzyme [77], Germain and colleagues reported a CHOP-10-independent UPR^mt^ model in which protein aggregates accumulated within the internal membrane space (IMS) when a mutant (catalytically inactive enzyme) form of endonuclease G (N174A) was overexpressed in breast adenocarcinoma MCF-7 cells [78].

##### Accumulation of Unfolded Proteins within the Mitochondrial Matrix

Pioneering work by Hoogenraad and collaborators first showed the selective induction of mitochondrial chaperones HSPD1 and HSPE1 in response to complete mitochondrial genome depletion (ρ*°*) in rat hepatoma H4 cells [60]. Later on, they set up a UPR^mt^ model based on the overexpression of a truncated form of the mitochondrial ornithine carbamoyltransferase (OTC) enzyme (OTC∆). The truncated form of the enzyme, while correctly localized in the organelle matrix, formed aggregates associated with the insoluble fraction after detergent extraction [79]. First clues of a UPR^mt^ have been described in the monkey kidney COS-7 cell line, as aggregated protein elimination correlates with the up-regulation of HSPD1 and ClpP. Therefore, OTC∆ abundance decreases when chaperones and proteases are overexpressed, yet abundance of the wild type OTC remains stable. In addition, co-immunoprecipitation experiments have also shown that HSPD1 and ClpP were bound to OTC∆ but not to its wild type counterpart [79].

Hoogenraad and colleagues also used reporter plasmids to highlight the induction of several genes that encoded mitochondrial proteins in response to OTC∆ aggregation such as chaperones (HSP60, HSP10, DnaJ homolog subfamily B member 1/HSP40, mitochondrial-processing peptidase subunit β, mitochondrial thioredoxin/MTRX), proteases (ClpP, ATP-dependent zinc metalloprotease YME1L1), and other mitochondrial proteins (Tim17, NADH dehydrogenase (ubiquinone) 1β subcomplex subunit 2/NDUFB2, caspase recruitment domain-containing protein 12/CARD12, endonuclease G, cytochrome C reductase) [80]. However, it is important to emphasize that increased expression of most of these target genes using artificial reporters has not been confirmed for endogenous-related genes [81] (personal unpublished data)—an observation that could be explained by differential epigenetic regulation and chromatin remodeling between a naked DNA promoter in the reporter construct and the DNA of authentic endogenous promoters [81]. In addition, the response seems to be specific; no change has been detected in the abundance for the UPR^er^ gene markers (BiP/GRP-78/78 kDa glucose-regulated protein, endoplasmin/GRP-94/94 kDa glucose-regulated protein), for cytosolic chaperones (Heat shock cognate 71 kDa protein, heat shock-related 70 kDa protein 2), and the requirement for mitochondrial localization. Demonstrated as the induction of nuclear gene encoding, mitochondrial proteins were not observed when cells were transfected with OTC∆ lacking the mitochondrial signal peptide [77,79].

Since these works, few advances have been made in the comprehension of mechanisms by which a UPR^mt^ is set up in mammals. However, the pathological relevance of the protein kinase RNA-activated (PKR) and the UPR^mt^ emerged in two murine models of colitis as well as in patients with inflammatory bowel diseases (IBDs) [82]. The expression of PKR increases in Mode K intestinal epithelial cells that overexpress the truncated form of OTC (OTC∆). Silencing experiments pointed out that PKR might play a role in the early phase of the UPR^mt^; the silencing of the kinase expression using siRNA prevents the phosphorylation of c-Jun and the induction of CHOP-10 [82]. In addition, these authors showed a PKR-dependent phosphorylation of eIF2α, suggesting the concomitant induction of ISR and UPR^mt^, as previously described in *C. elegans* [82]. Various strategies were used to disrupt the stoichiometric equilibrium between nuclear- and mitochondrial-encoded ETC (Electron Transport Chain) subunits to trigger a UPR^mt^. Among them, doxycycline, an inhibitor of mitochondrial translation, triggered the activation of a UPR^mt^ activation in AML12 cells (a mouse hepatic cell line), as demonstrated by an increased expression of HSPD1 protein and the activation of a reporter gene driven by the ClpP promoter [45]. Similar results were obtained when mitochondrial protein abundance was increased by either increasing the NAD^+^ concentration with two precursors of its synthesis (nicotinamide mononucleotide (NAM) and nicotinamide riboside (NR)) known to trigger the expression of several nuclear gene encoding mitochondrial proteins in an NAD-dependent protein deacetylase Sirtuin-1 (SIRT1)-dependent manner [83] or by boosting mitochondrial biogenesis by mTOR inhibition with rapamycin [67]. In primary murine hepatocytes, it was also confirmed that an antioxidant response mediated by superoxide dismutase 2 (SOD2) was associated with the UPR^mt^, and that both responses were dependent on the Sirtuin-1 deacetylase [67]. Interestingly, at least one other Sirtuin is involved in the resolution of mitochondrial proteotoxic stress. Indeed, NAD-dependent protein deacetylase Sirtuin-3 (SIRT3) has been shown to be activated in response to a large panel of mitochondrial stresses known to induce the accumulation of protein aggregates and/or ROS, including antimycin A, rotenone, an inhibitor of heat shock protein HSP90 and the overexpression of mutated EndoG (ΔMLS-Endo G-His), mutated SOD1, and OTC∆. These mitochondrial stresses activated an antioxidant response and mitophagy in a SIRT3-dependent manner, but the possible SIRT3-dependency of the UPR^mt^ (evaluated by CHOP-10 and HSP60 mRNA levels) was not reported. Interestingly, the proteotoxic stress resulted in a heterogeneous mitochondrial phenotype, a fraction of the mitochondria under severe stress. The cell viability under proteotoxic stress was compromised in the absence of SIRT3. Altogether, this set of data suggests that cells undergoing proteotoxic stress present a heterogeneous adaptive response with the induction of a UPR^mt^ to resolve the stress in mildly affected mitochondria and a SIRT3-orchestrated mitophagy to remove irreversibly damaged organelles [84].

Despite the requirement for HAF-1 to observe the activation of a UPR^mt^ in *C. elegans*, no clear mammalian orthologue of this protein has been identified thus far. However, Rainbolt and colleagues confirmed that, in mammalian cells, the expression of the worm Tim17 mutant and the reduction of mitochondrial imported induce UPR^mt^. In addition, the knock down of Tim17 in HEK293 cells resulted in a slight increase in HSPD1 and YME1L1 mRNA abundance [74]. Interestingly, these authors proposed a stress pathway based on the phosphorylation of eIF2α and the attenuation of translation that leads to a down-regulation of Tim17 abundance, which is further enhanced by an increased YME1L1-dependent degradation of the protein. Ultimately, a lower abundance of Tim17 and, as a consequence, the reduced mitochondrial protein import might thus facilitate the activation of the UPR^mt^ [74].

As for HAF-1, no mammalian orthologue of ATFS-1 has been identified to date. Detailed mechanisms as well as several actors of the UPR^mt^ are thus still likely missing in mammals, especially regarding the effectors involved in the transcriptional regulation of target genes. However, both DVE-1 and UBL5 have mammalian orthologues: SATB2 and UBL5, respectively [68]. Interestingly, SATB2 is a global chromatin organizer, and this protein is also able to form a complex with UBL5; however, no evidence of involvement of SATB2/UBL5 in the mammalian UPR^mt^ has been experimentally demonstrated thus far [68].

The analysis of the HSPD1/HSPE1 bidirectional promoter revealed the existence of a CHOP-10-C/EBPβ binding element crucial for the activation of the UPR^mt^-responsive genes. In addition, the abundance of these two transcription factors increased upon the overexpression of OTC∆ [79,80]. As in *C. elegans*, the current mammalian model of the UPR^mt^-induced gene expression would be a two-step process. It would firstly require the expression of transcription factors, such as CHOP-10, which would in turn activate the expression of gene encoding of mitochondrial chaperones/chaperonins and proteases.

In terms of upstream signaling pathways, it seems that the phosphorylation of c-Jun by JNK2 and/or PKR is necessary to activate the expression of these transcription factors [77,82]. Phosphorylated c-Jun would bind to an AP-1 binding site within the CHOP-10 promoter, allowing the regulation of a UPR^mt^-specific gene expression. These genes do not contain the well-described endoplasmic reticulum stress response element (ERSE) required for the classic genes regulated during the UPR^er^ [80]. However, considering the multiple cell responses involving CHOP-10 and the putative 3522 promoters of nuclear genes predicted to contain the binding consensus element for this transcription factor, it is likely that additional factors are required to specifically regulate gene expression during the UPR^mt^ [80]. The sequence alignment of the 1000 bp promoter sequences of the UPR^mt^-induced genes revealed two well-conserved elements (except for the HSPD1/HSPE1 bidirectional promoter) surrounding the CHOP-10 binding site at a constant distance, named MURE1/2 (Mitochondrial unfolded protein response element 1/2) [80]. The requirement of these regulatory elements was demonstrated by reporter plasmid experiments as mutations within MURE1 or MURE2 to reduce the transcriptional activity of the UPR^mt^-target genes triggered by the overexpression of OTC∆ [80]. However, the relevance of the MURE1-CHOP-10-MURE2 element might be questionable because (i) it has not been confirmed since then; (ii) this motif was widespread in the genome and was not restricted to the UPR^mt^ responsive genes (personal unpublished data); and (iii) the ligands putatively associated with these two regulatory elements have not yet been identified.

##### IMS-Associated mtUPR

In addition to the UPR^mt^ initiated by events occurring in the mitochondrial matrix, Germain and Papa described another UPR^mt^ model triggered by mitochondrial IMS protein aggregate accumulations, which caused the overexpression of a mutant form of endonuclease G (EndoG-N174A) in breast cancer MCF-7 cells, a condition that increases HTRA2/OMI protein abundance (a mitochondria-located serine protease that can be released by mitochondria during apoptosis) [85], NRF1 expression, and enhanced proteasome activity [78]. To contrast with OTC∆-induced UPR^mt^, the IMS stress response is not dependent on CHOP-10 expression, but rather relies on the ligand-independent activation of estrogen receptor α (ERα). It has been clearly shown that the overexpression of EndoG-N174A induces the RAC-α serine/threonine-protein kinase/AKT/PKB-dependent phosphorylation of ERα phosphorylation (Ser167). Additionally, AKT activation is dependent on ROS production triggered by the IMS stress as a treatment, with the antioxidant *N*-acetyl cysteine (NAC), completely abolishing the activation of ERα [78]. More recently, this research group deciphered events associated with IMS stress in triple negative cancer cells (lacking ERα) and highlighted the involvement of the mitochondrial SIRT3 in the regulation of the antioxidant response and mitophagy [84]. Strikingly, while these authors had previously shown that EndoG-N174A overexpression in MCF-7 cells did not induce a cell signaling response comparable to the one observed in response to the accumulation of truncated OTC in the mitochondrial matrix, in ERα-deficient breast cancer cells, they recently revealed that IMS stress also increases the expression of CHOP-10 and HSPD1 [84].

In conclusion, it seems that different retrograde responses that are dependent on the mitochondrial compartment and location at the origin of the stress could be initiated. Indeed, as we have seen, different quality control factors have been described, depending on the organelle compartment, such as ClpP and HSPD1, for matrix proteins or serine protease HTRA2/Omi and the proteasome for IMS proteins. Thus, because initiating stress and responding effectors to recover protein homeostasis are different between the matrix and IMS stress, this may explain the existence of independent signaling pathways such as those described with CHOP-10 and OTC∆, or ERα and EndoG-N174A. In a recent study, we showed that, the transcription factor CHOP-10 was systematically overexpressed when the expression of mtDNA was inhibited by different means and in different cell types, while the UPR^mt^-related gene markers HSPD1 and ClpP were not induced [86]. Furthermore, increased expression of this transcription factor correlated with the activation of another stress-responsive pathway called the integrated stress response (ISR) [86].

### 1.3. The Integrated Stress Response (ISR)

Although the integrated stress response (ISR) is not usually described as a typical retrograde response, several studies reported its activation in response to mitochondrial dysfunction [86,87,88,89,90]. The next paragraph presents the main features of ISR as well as its crosstalk with mitochondrial stress because it is a complementary pathway to the UPR^mt^.

**Figure 2:**
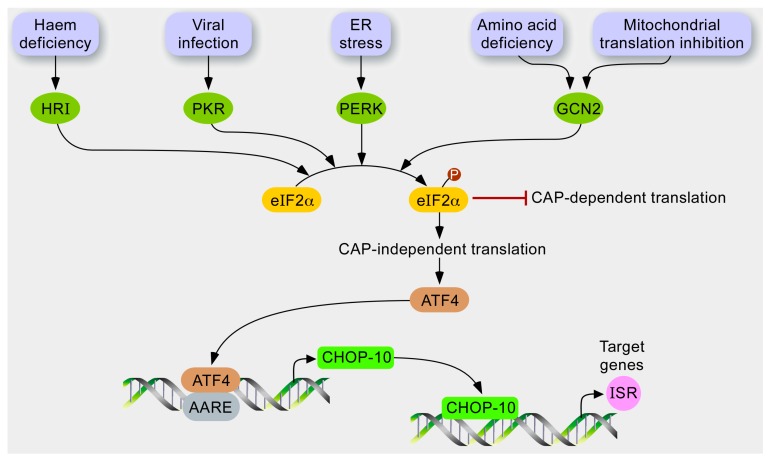
Hypothetical model for stress response to mitochondrial dysfunction. In response to various stresses, several kinases, such as HRI, PKR, PERK, and GCN2, are activated and converge to phosphorylate the translation initiation factor eIF2α. In turn, the phosphorylation of eIF2α has at least two consequences. While the cytosolic translation is globally attenuated as it inhibits CAP-dependent translation, mRNAs containing a uORF are preferentially translated, such as the transcription factor ATF4, which controls the expression of CHOP-10. The profit of this GCN2-eIF2α-ATF4 pathway might be stress attenuation gained by relieving the load of proteins imported into the mitochondria and increasing the expression of integrated stress responsive genes.

The ISR is a stress adaptive pathway conserved by evolution as recovered from yeast to human. In mammals, molecular effectors of this signaling pathway (Figure 2) are activated during ER stress, amino acid depletion [91], virus infection, oxidative stress, heme deprivation, or UV irradiation [92].

These stressors are sensed by four different kinases: Eukaryotic translation initiation factor 2-α kinase 1/heme-regulated inhibitor (HRI), PKR, RNA-dependent protein kinase-like endoplasmic reticulum kinase (PERK), and general control non-derepressible 2 (GCN2) that converge to the phosphorylation of eIF2α eukaryotic translation Initiation Factor 2α at serine 51 [93]. Phosphorylation of eIF2α is a central event of the ISR; it integrates upstream signals to reduce the ATP-consuming events, such as cytosolic translation, and to regulate the specific expression of a set of nuclear genes. Phosphorylated eIF2α (P-eIF2α) inhibits recycling of its GTP-bound form (active) by eIF2B, which is required for delivery of the methionyl-tRNA to the ribosome during translation initiation [94]. In addition, P-eIF2α also elicits preferential translation of mRNAs containing an upstream open reading frame (uORF) in their 5ʹ-leader sequence such as the bZIP transcription factor activating transcription factor (4ATF4). Under unstressed conditions, translation initiation occurs at the start codon of the first uORF (uORF1) within the 5ʹ-leader of ATF4 mRNA. While the ribosome scans the sequence, after translation termination at the uORF1, the ribosome does not dissociate from the mRNA and resumes scanning to reach uORF2. When eIF2α is not phosphorylated, it is rapidly recycled in its eIF2α-GTP form—a reaction that allows rapid re-initiation of translation at the site. However, the inhibitory uORF2 is out of frame from the ATF4 coding region, and the ribosome dissociates without translation of ATF4. When cells are under stressful conditions, the availability of eIF2α-GTP decreases and after termination at uORF1, the ribosome will scan through uORF2 because there is not enough eIF2α-GTP to re-initiate translation in a rapid manner. As the ribosome scans to reach the coding sequence of ATF4, eIF2α-GTP is sufficiently recycled to initiate translation at the start codon [92].

ISR also regulates gene expression by an interconnected set of transcription factors, such as ATF3 and CHOP-10, which are both induced by ATF4. Together with other bZIP transcription factors, they regulate many other downstream genes, such as homocysteine-responsive endoplasmic reticulum-resident ubiquitin-like domain member 1 (HERPUD1) and tribbles homolog 3 (TRIB3), which first help to either resolve the stress or, alternatively, to induce apoptosis if the stress is too severe or persists for too long [91]. The protein encoded by HERPUD1 is a protective protein that participates in the endoplasmic reticulum-associated degradation (ERAD) pathway during ER stress [95]. Interestingly, these authors showed that HERPUD1 might be neuroprotective during ER stress by stabilization of Ca^2+^ storage and maintenance of mitochondria function [95]. The tribbles homolog 3 protein is a pseudo-kinase because, despite having a region of high homology with kinase domains, it lacks a consensus sequence for protein phosphorylation. So far, TRIB3 is known to directly interact with different proteins, such as AKT, C/EBPs (CCAAT/enhancer binding proteins), and ATF4/CHOP-10, thereby regulating the biological process and responses such as insulin signaling, autophagy, adipogenesis, and induction of apoptosis, respectively [96].

The ISR is interconnected with several pathways, and some effectors are shared by other signaling such as the UPR^er^ or the UPR^mt^ [82]. In addition, the GCN2 kinase can be activated by amino acid depletion, glucose depletion, UV irradiation, proteasome inhibition, and oxidative stress [93]. The mechanism of activation upon amino acid starvation is well described. Under normal conditions, the kinase is kept inactive by auto-inhibitory molecular interactions. Upon binding of unloaded tRNA to the histidyl-tRNA synthetase-like domain, an allosteric rearrangement creates an open conformation of the protein, and GCN2 is activated by auto-phosphorylation (Thr898 and Thr903) within the activation loop [97].

This kinase is a good example to illustrate divergent signaling pathways of the cell responses following eIF2α phosphorylation. Indeed, while the signaling pathway downstream the P-eIF2α triggered by the UV-irradiation enhances the activation of NF-κB and cell survival, the phosphorylation of eIF2α in response to the inhibition of proteasome leads to the expression of CHOP-10 [98,99]. As mentioned, CHOP-10 is a pleiotropic transcription factor that can form heterodimers with other members of the bZIP family transcription factors, such as C/EBPs and ATF4, to function either as a trans-activator or a trans-repressor of target genes [100,101,102]. Moreover, while CHOP-10 has been associated with apoptosis in many different conditions, paradoxically, recent studies have revealed that it can also promote cell survival during ER stress and amino acid starvation by regulating the expression of several autophagy-related genes such as p62, ATG5, ATG7, and ATG10 [103,104]. The resulting pro- or anti-apoptotic response is most likely dependent on the integration and balance of multiple signals that are activated in parallel with the cell nuclear background, together with the type/origin, nature, severity, and duration of the stress.

In addition to this coordinated expression of chaperones and proteases, when the defect is too severe, the next level of quality control is the degradation of the entire organelle by specific autophagy called mitophagy [105]. We have seen clearly that mitochondria display several lines of quality control mechanisms: mitochondria-specific chaperones and proteases protect against misfolded proteins at the molecular level, and fission/fusion and mitophagy segregate and eliminate damage at the organelle level [9]. Therefore, if an increase in unfolded proteins in mitochondria activates a UPR^mt^ to increase chaperone production, the mitochondrial serine/threonine-protein kinase PINK1 and the E3 ubiquitin-protein ligase parkin, whose mutations cause familial Parkinson’s disease, remove depolarized mitochondria through mitophagy [106]. This research group also showed that both phenomenon were linked; the expression of unfolded proteins in the matrix causes the accumulation of PINK1 on energetically healthy mitochondria, resulting in mitochondrial translocation of Parkin and mitophagy and subsequent reduction of unfolded protein load, a response that is strongly enhanced by the knockdown of the mitochondrial Lon protease homolog [105].

### 1.4. What Is the (Patho)Physiological Relevance of a UPR^mt^ in Mammals?

#### 1.4.1. The UPR^mt^ and Neurodegenerative Diseases

The accumulation of unfolded or aggregated proteins is a hallmark of a number of neurodegenerative diseases, including Alzheimer’s disease (AD) and Parkinson’s disease (PD), although it is currently unclear if these protein aggregates participate or are the consequence of such pathologies. For instance, extracellular amyloid β deposits are characteristic of AD, but they are also present in mitochondria [107]. Amyloid β precursor protein is cleaved by the mitochondrial HTRA2/Omi serine protease [108] and the latter has been shown to delay the aggregation of the amyloid β 1–42 peptide [109]. Although HTRA2 is a target gene of IMS-induced UPR^mt^ (Figure 1), a putative role of the UPR^mt^ in this pathology has not been demonstrated clearly. Regarding PD, accumulation of α-synuclein has been demonstrated in the mitochondria of post-mortem brains of PD patients as well as in cell models of PD [110,111]. Interestingly, a higher level of misfolded respiratory proteins was detected in post-mortem brains of PD patients, which correlates with an elevated abundance of HSP60 [112], suggesting the occurrence of a UPR^mt^. However, the UPR^mt^ is still poorly characterized in PD models, contrary to mitophagy, because PINK and/or Parkin deficiencies are strongly associated with PD [113]. Clearly, investigating the relationship between the UPR^mt^ and neurodegenerative diseases would be of great interest. In addition, the development of new tools to better monitor the UPR^mt^ *in vivo* in individual cells would be welcome, as the mitochondrial proteotoxic stress generates a heterogeneous phenotype associated with a UPR^mt^ and/or mitophagy [84].

#### 1.4.2. The UPR^mt^ in Aging

One of the pioneering studies reporting a link between aging and the UPR^mt^ was the silencing of cco-1, a subunit of Cytochrome c oxidase, in neuronal tissue of *C. elegans*, which increases lifespan and induces a UPR^mt^ not only in neurons, but also in the intestine [55], suggesting an inter-tissue signaling that results in a UPR^mt^ activation. Since then, the UPR^mt^ has been implicated in increased lifespan in worms, flies, and mice, highlighting the importance of maintaining mitochondrial proteostasis for longevity. This aspect will not be developed here; several reviews have been recently devoted to UPR^mt^ and aging [56,62,114].

#### 1.4.3. The UPR^mt^ and Stem Cells

Recently, Mohrin and co-workers highlighted a SIRT7-NRF1 regulatory axis in the UPR^mt^ and linked the UPR^mt^ to hematopoietic stem cells’ (HSC) quiescence and aging [115]. They first demonstrated that the NAD^+^-dependent deacetylase SIRT7 is induced in response to a UPR^mt^. SIRT7 directly binds to the transcription factor NRF1, specifically on the promoter of gene encoding mitochondrial ribosomal proteins and mitochondrial translation factors, to reduce, indirectly, their expression. SIRT7 induction thus contributes to attenuating the mitochondrial translation, thereby decreasing the proteotoxic stress imposed on the organelle. In addition, SIRT7 deficiency results in a constitutive UPR^mt^. Because stem cells undergo an oxidative metabolic shift when entering into differentiation, it is not surprising that SIRT7, by limiting mitochondria biogenesis and the UPR^mt^, contributes to HSCs’ quiescence and prevents differentiation. Furthermore, the activation of a UPR^mt^ observed in aged HSCs has been linked to SIRT7 decreasing in these cells, contributing to their decline. Interestingly, reintroduction of SIRT7 in aged HSCs reduces the UPR^mt^ and improves their regenerative capacities. Altogether, these authors concluded that the UPR^mt^ might be an aged-sensitive metabolic checkpoint to regulate HSCs’ renewal properties.

#### 1.4.4. The UPR^mt^ in Innate Immunity

Several bacterial pathogens target the mitochondria, disrupting essential functions, such as calcium and redox homeostasis, or mitochondrial morphology [116]. However, the impact of pathogens on the UPR^mt^ is still poorly described, especially in mammals. In *C. elegans*, several bacteria strains, and particularly *P. aeruginosa*, activate a UPR^mt^, as shown by a specific chaperone induction in the intestine [117]. Because the worm is more susceptible to *P. aeruginosa* exposure when ATFS-1 expression is down-regulated, the UPR^mt^ might be protective against bacterial infection. In addition, the knockdown of an mtDNA-encoded ATP synthase subunit not only activates the UPR^mt^, but also the expression of innate immune genes and makes the worm more resistant to *P. aeruginosa* exposure. Finally, these authors showed that human cells undergoing a UPR^mt^ also induce the transcription of antimicrobial peptides (reviewed in [113]). In line with these observations, PKR-mediated UPR^mt^ has been highlighted in two murine models of colitis, as well as in patients with inflammatory bowel diseases under inflammatory conditions [82]. The UPR^mt^ might thus represent one of the mechanisms that senses bacterial pathogens in the intestinal mucosae and adapts the cell functions and immune response (reviewed in [118]). However, it is still currently unclear whether PKR-dependent induction of a UPR^mt^ in colitis models is detrimental or beneficial due to controversial data found in the literature [82,119].

Importantly, the sensitivity to the UPR^mt^ may vary according to different parameters, including tissue-specificity and stress intensity. This has been demonstrated by Dogan and co-workers, who studied a mouse model that lacked a protein directly involved in mitochondrial translation, such as the gene DARS2 encoding the mitochondrial aspartate-tRNA ligase, specifically in heart and skeletal muscle tissues [120]. While both tissues presented a comparable mitochondrial respiratory deficiency, the strategies developed to cope with this proteotoxic stress were different, depending on the tissue. DARS2-deficient hearts from 6-week-old mice presented a mitochondrial cardiopathy, with an increase in mitochondria biogenesis and activation of a UPR^mt^, accompanied by reduced autophagy markers. However, 3-week-old mice showed no sign of cardiopathy, suggesting that the mitochondrial stress imposed by the conditional loss of DARS2 had not reached a sufficient level to trigger these stress responses. Conversely, these stress responses were not observed in the skeletal muscles of 6-week-old mice despite a 60% to 80% decrease in the activity of respiratory chain complexes, presumably because a low turnover of mitochondrial transcripts was the preferred strategy to cope with mitochondrial stress in this tissue [120].

## 2. Conclusions

### 2.1. The UPR^mt^ versus ISR or Both in Response to a Mitochondrial Stress?

While the number of papers reporting on the UPR^mt^ is ever growing, it is interesting to note that this stress response is still poorly defined in terms of target genes and signaling pathways, especially in mammalians. This is probably reinforced by the fact that some researchers tend to designate UPR^mt^ as any kind of retrograde response triggered by a mitochondrial stress, even if the stress has not been associated with the accumulation of unfolded protein aggregates. Although the pioneering work performed by the group of Hoogenraad [79] established a large list of target genes specifically induced by the UPR^mt^ (and not by the UPR^er^), these interesting findings were mainly obtained with reporter assays. Only a few of these target genes were shown later to be induced at the endogenous level by one or several of the mitochondria stressing conditions listed in Figure 1. These endogenous UPR^mt^ targets were CHOP-10 [79], HSP60 [78,79,82,115], ClpP [115], and YME1L1 [74], although the last three UPR^mt^ markers were generally induced in a modest manner. However, it is interesting to point out that other types of mitochondrial stresses, such as the inhibition of mitochondrial translation or the depletion of mtDNA [86], MELAS and NARP mtDNA point mutations [88], inhibition of the respiratory chain by rotenone and antimycin A [89], or loss of HTRA2 expression [90] do not induce HSP60 and ClpP expression, but rather activate the integrated stress response (ISR) characterized by an attenuation of the cytosolic translation achieved through the phosphorylation of eIF2α and by the induction of another set of target genes including CHOP-10, TRIB3, and HERPUD1 [86]. Importantly, Rainbolt and co-workers revealed that arsenite, an ETC inhibitor, triggered the ISR, with a transient phosphorylation of eIF2α, accompanied by the induction of HSP60 and Tim17 at the transcript level, but not at the protein level [74]. Tim17 protein abundance strongly decreased in response to arsenite due to both a decreased biogenesis and an increased YME1L1-dependent degradation, thereby reducing the mitochondrial protein import. Interestingly, depletion of Tim17 by RNA interference not only reduced mitochondrial import, but also modestly increased (1.5-fold) the transcript level of HSP60 and YME1L1 [74]. Altogether, this suggests a two-wave response to the arsenite-induced mitochondrial stress: the first wave activated the ISR, with a transient phosphorylation of eIF2α that reduces Tim17 synthesis. The lower mitochondrial import capacity associated with reduced Tim17 abundance in turn induced the transcription of some UPR^mt^ target genes. It is important to know whether cooperation between the ISR and the UPR^mt^ activation could be generalized to other types of mitochondrial stresses, which still needs to be addressed, although Haller’s group has already shown that the UPR^mt^ observed in human intestinal epithelial cells overexpressing OTC∆ is dependent on PKR-mediated ISR [82]. Researchers interested in mitochondria-induced stress responses should therefore be encouraged to monitor the kinetics—systematically—of both the UPR^mt^ and the ISR markers, including the efficiency of the mitochondrial import capacity.

It is important to emphasize the fact that, while the ISR and the UPR^mt^ were first discriminated based on the source of mitochondrial stress generated (depletion of mtDNA *versus* accumulation of misfolded proteins) in experimental models, it is most likely that a certain level of overlap exists between these two signaling pathways, especially as several effectors are common to both cell responses. One can easily imagine that mtDNA depletion and mutations affecting ETC that are known to trigger ISR could also eventually lead to ROS-dependent oxidation of mitochondrial proteins and lipids, causing defects in folding or misassembling of mitochondrial protein complexes, initiating a UPR^mt^.

### 2.2. Future Directions

Important questions remain unanswered regarding the UPR^mt^, as well as mitochondria-induced ISR. First, while the link between a mitochondria proteotoxic stress and the cytosolic UPR^mt^ cascade is relatively clear in *C. elegans*, it remains poorly understood in mammals. Does the mammalian UPR^mt^ signaling depend on peptide efflux? If so, what are the peptide transporters and the primary cytosolic targets?

Second, the attenuation of cap-dependent translation, achieved through phosphorylation of eIF2α, is a shared and common feature between the UPR^er^, the UPR^mt^, and the ISR (Figure 1 and Figure 2). These three pathways are powerful mechanisms for alleviating the proteotoxic stress imposed on organelles or cytosol. However, an intriguing feature is the kinetics of eIF2α phosphorylation: while transient in the case of a UPR^er^, it is sustained (up to 30–48 h) in the case of a UPR^mt^ triggered by ∆OTC overexpression [82], and in the case of ISR, it is induced by the inhibition of mitochondrial translation [86]. In response to a UPR^er^, it is known that the induction of encoding the protein phosphatase 1 regulatory subunit 15A/Growth arrest and DNA damage-inducible protein (PPP1R15A/GADD34) contributes to dephosphorylation of the phosphorylated form of eIF2α and thus resumes cap-dependent translation. One might wonder how cells deal with a reduced cap-dependent translation for as long as 2–3 days, a phenomenon accompanied by cell growth arrest in some cell types, but not all of them (Sébastien Michel, University of Namur, Belgium. Personal communication, 2014). During this period, several ISR-target genes are induced. Even if the presence of internal ribosome entry sites (IRES) has been described for at least some of them, one cannot exclude a specific regulation at the translation level on these ISR-responsive transcripts, following the mechanism of RNA regulon control [121]. In line with this hypothesis, a recent multi-layered “omics” dissection of mitochondrial activity in the liver proteome from 40 strains of the BXD mouse genetic reference population revealed that six members of the UPR^mt^ pathway are coordinately regulated; this occurs more strongly at the protein level than at the transcript level [122].

Third, the specificity of CHOP-10-dependent cell responses needs to be investigated. Indeed, CHOP-10 is induced in response to number of cell stress conditions, such as nutrient deprivation, viral infection, hypoxia, or accumulation of protein aggregates in organelles such as the endoplasmic reticulum and mitochondria. This transcription factor is undoubtedly a key actor for coordinating the adequate cell response that ranges from apoptosis, the UPR^er^, and the UPR^mt^ to the ISR. In addition, CHOP-10 was also shown to reduce the proliferation of intestinal cells in models of acute and chronic colitis [123]. However, the molecular determinants that confer the specificity of CHOP-10-dependent stress-response are still largely unclear and might involve specific post-transcriptional modifications of CHOP-10 as well as the activation of additional transcriptional regulators.

Fourth, while inducing chaperones and proteases to restore proteostasis makes sense, the biological significance of some ISR target genes in the context of a mitochondrial proteotoxic stress remains undetermined. For instance, it has been shown that the induction of TRIB3 participates in a negative feedback loop to repress most of the ATF4-dependent ISR target genes [124]. However, the biological role of other ISR-induced genes, and particularly the induction of several aminoacyl-tRNA synthetases in response to mitochondrial stresses [88,124], still requires analysis. Briefly, in all eukaryotes, aminoacyl-tRNA synthetases are sequestrated in large multimeric complexes, where they ensure their primary role of tRNA aminoacylation. It has been shown that upon release from these complexes, they can carry out alternative functions. When yeasts are submitted to the diauxic transition from anaerobic glycolysis to oxidative respiration, two aminoacyl-tRNA synthetases, cERS and cMRS, dissociate from their cytosolic anchor complex, relocating to the mitochondria and the nucleus, respectively. While cERS stimulates mitochondrial translation, cMRS triggers the transcription of nuclear gene encoding of ATP synthase subunits, enabling synchronization of the expression of mitochondria-encoded and nuclear-encoded subunits of the complex V [125]. The fact that non-canonical functions have been recently highlighted for two tRNA synthetases in the yeast could pave the way for new researches needed in the future to fully understand signaling and effectors of the UPR^mt^ in mammalians.
